# The crosstalk between immune cells and tumor pyroptosis: advancing cancer immunotherapy strategies

**DOI:** 10.1186/s13046-024-03115-7

**Published:** 2024-07-10

**Authors:** Mengyuan Hu, Fengying Deng, Xinlei Song, Hongkun Zhao, Fei Yan

**Affiliations:** 1 Department of Pathology and Pathophysiology, Faculty of Basic Medical Sciences, Kunming Medical University, Chenggong District, 1168 Chunrong West Road, Yunhua Street, Kunming, 650500 Yunnan China; 2 Key Laboratory of Yunnan Province, Yunnan Eye Institute, Affiliated Hospital of Yunnan University, Yunnan University, 176 Qingnian Road, Wuhua District, Kunming, 650031 Yunnan China

**Keywords:** Pyroptosis, Tumor, Immune cells, Tumor immune microenvironment, Immunotherapies

## Abstract

Pyroptosis is a cell death process characterized by cell swelling until membrane rupture and release of intracellular contents. As an effective tumor treatment strategy, inducing tumor cell pyroptosis has received widespread attention. In this process, the immune components within the tumor microenvironment play a key regulatory role. By regulating and altering the functions of immune cells such as cytotoxic T lymphocytes, natural killer cells, tumor-associated macrophages, and neutrophils, tumor cell pyroptosis can be induced. This article provides a comprehensive review of the molecular mechanisms of cell pyroptosis, the impact of the tumor immune microenvironment on tumor cell pyroptosis, and its mechanisms. It aims to gain an in-depth understanding of the communication between the tumor immune microenvironment and tumor cells, and to provide theoretical support for the development of new tumor immunotherapies.

**Supplementary Information:**

The online version contains supplementary material available at 10.1186/s13046-024-03115-7.

## Introduction

Pyroptosis, a form of programmed cell death, is closely connected to the inflammatory response, facilitating communication between innate and adaptive immunity [1, 2]. This emerging type of regulated cell death significantly influences cancer modulation, antitumor immunity, and the prognosis of cancer patients [2]. The impacts of pyroptosis are not only inhibiting tumor cell proliferation but also shaping an immunosuppressive microenvironment whichs promote tumor growth [3]. This immunosuppressive microenvironment has implications for the efficacy of anticancer therapy.

The tumor microenvironment (TME) comprises a diverse array of non-tumor cells, such as immune cells, stromal cells, and blood vessels, as well as structural components within the tumors, including extracellular matrix proteins and cytokines. These components interact with the tumor cells, collectively influencing tumor development, metastasis, and ultimately determining the tumor's responsiveness to various treatment strategies. Cancer cells often develop drug resistance due to genomic instability, while non-tumor cells in the TME are genetically more stable and respond better to therapies [4]. Targeting the TME is more effective than directly targeting cancer cells. As a part of the TME, the tumor immune microenvironment (TIME) which includes T lymphocytes, natural killer (NK) cells, tumor-associated macrophages (TAMs), and tumor-associated neutrophils (TANs), plays an important role in tumor development by helping tumor cells evade immune surveillance. Therapies that target immune cells in TIME to induce tumor cell pyroptosis can shift the TME to an immunostimulatory state, making them valuable for tumor immunotherapy [5]. Reshaping the TIME and restoring the tumor-killing ability of anti-tumor immune cells is a key area of research. Tumor immunotherapy, particularly chimeric antigen receptor (CAR)-T cell therapy and immune checkpoint inhibitors (ICIs), has shown promising results in combating tumor immune escape [6].

Pyroptosis is a critical factor in the origin, management, and outcome of tumors. Understanding the features and molecular mechanisms of cell pyroptosis, as well as the regulatory impact of TIME on tumor cell pyroptosis, is essential for advancing therapeutic approaches and improving treatment efficacy. This article aims to explore the characteristics and molecular mechanisms of pyroptosis, the influence of immune cells within the TIME on tumor cell pyroptosis, and two key tumor immunotherapy approaches. By gaining a comprehensive understanding of pyroptosis, this research aims to provide valuable insights for the development of new tumor immunotherapy strategies.

## The characteristics and molecular mechanisms of cell pyroptosis

### The emerging of pyroptosis

**Fig. 1:**
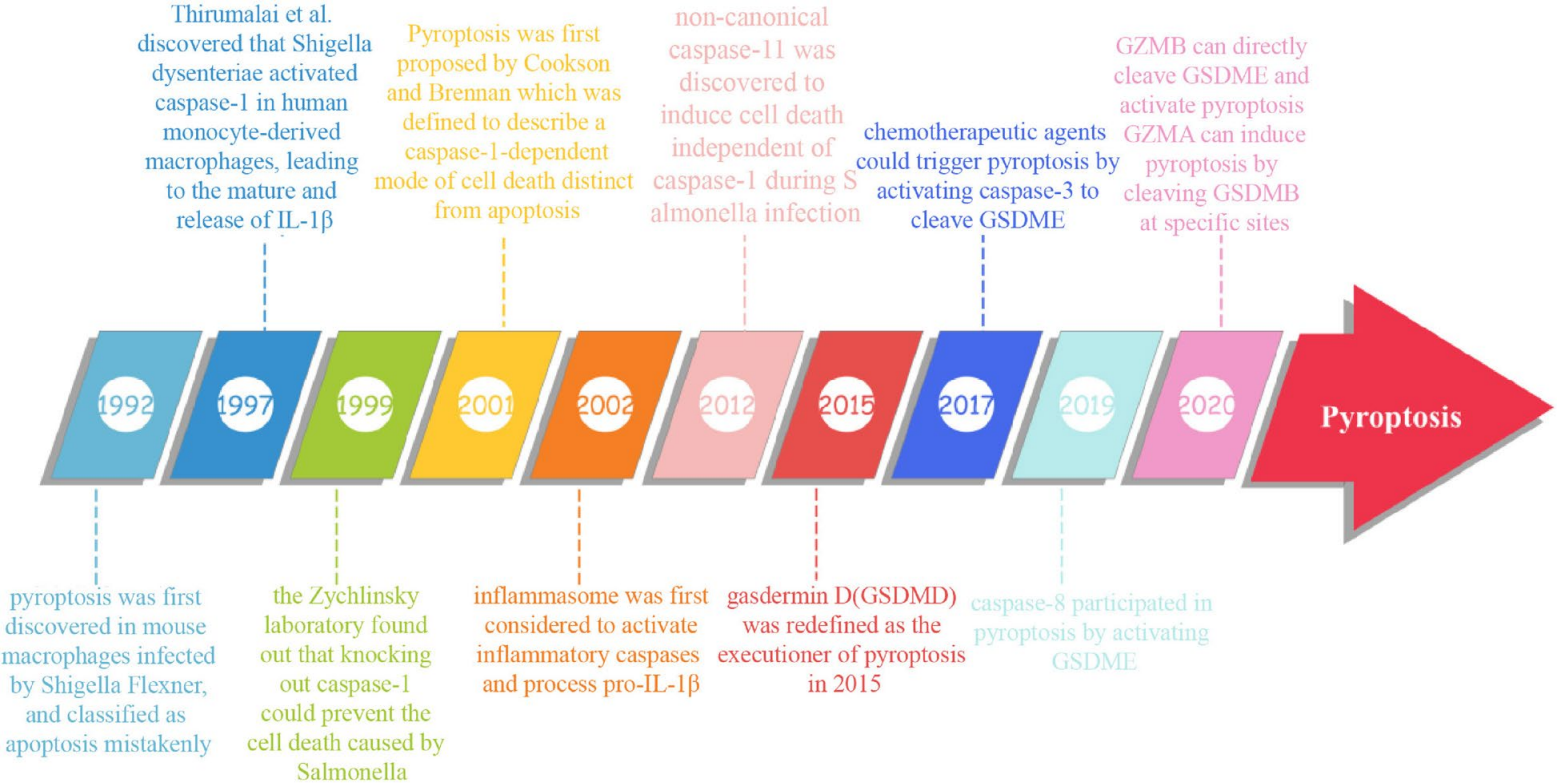
Time course of pyroptosis development

In the 1990s, pyroptosis was initially discovered in mouse macrophages infected by Shigella Flexner and was classified as apoptosis mistakenly [7, 8]. Subsequent research by Thirumalai et al. in 1997 revealed that Shigella dysenteriae activated caspase-1 in human monocyte-derived macrophages, leading to the maturation and subsequent release of Interleukin-1β (IL-1β) [9]. The Zychlinsky laboratory in 1999 demonstrated that knocking out caspase-1 could prevent cell death caused by Salmonella [10]. The term “pyroptosis”' was coined in 2001 by Cookson and Brennan, defining it as a caspase-1-dependent form of cell death distinct from apoptosis [11]. The concept of inflammasome activating inflammatory caspases and processing pro-IL-1β was introduced in 2002 [12]. In 2012, non-canonical caspase-11 was discovered to trigger cell death independently of caspase-1 during Salmonella infection [13]. (Fig. 1) gasdermin D (GSDMD) was redefined as the executioner of pyroptosis in 2015 [5, 14, 15]. Since then, other proteins in the gasdermin family have been found to mediate pyroptosis through caspase cleavage. Wang et al. and Rogers et al. demonstrated in 2017 that chemotherapeutic agents could induce pyroptosis by activating caspase-3 to cleave GSDME [16, 17]. The discovery has been widely utilized in tumor treatment. The Nomenclature Committee on Cell Death revised the definition of pyroptosis in 2018, describing it as a form of regulated cell death that critically depends on the formation of plasma membrane pores by members of the gasdermin protein family, often (but not always) as a consequence of inflammatory caspase activation. Notably, caspase-8 was found to participate in pyroptosis by activating GSDME in 2019 [18]. Furthermore, enzymes produced by immune cells have been identified as mediators of pyroptosis, working by recognizing and cleaving gasdermin proteins, thus shedding light on the intricate communication between the immune microenvironment and parenchymal cells. Reports from 2020 suggest that granzyme B (GzmB) can directly cleave GSDME, leading to the activation of pyroptosis and triggering the antitumor immune response [19]. Additionally, granzyme A (GzmA) in cytotoxic lymphocytes has been shown to induce pyroptosis by hydrolyzing GSDMB at specific sites, further advancing our understanding of pyroptosis [20].

In summary, pyroptosis is characterized by cell swelling, membrane rupture and the release of various pro-inflammatory factors such as IL-1β, Interleukin-18 (IL-18), ATP, and high mobility group box 1 protein (HMGB1) [21, 22]. Over time, researchers have gained a better understanding of the molecular mechanisms of pyroptosis, categorizing the pyroptosis pathway into canonical pyroptosis pathway, non-canonical pyroptosis pathway, other caspase-mediated pyroptosis pathways, and enzyme-induced pyroptosis pathways.

### Canonical pyroptosis pathway

**Fig. 2:**
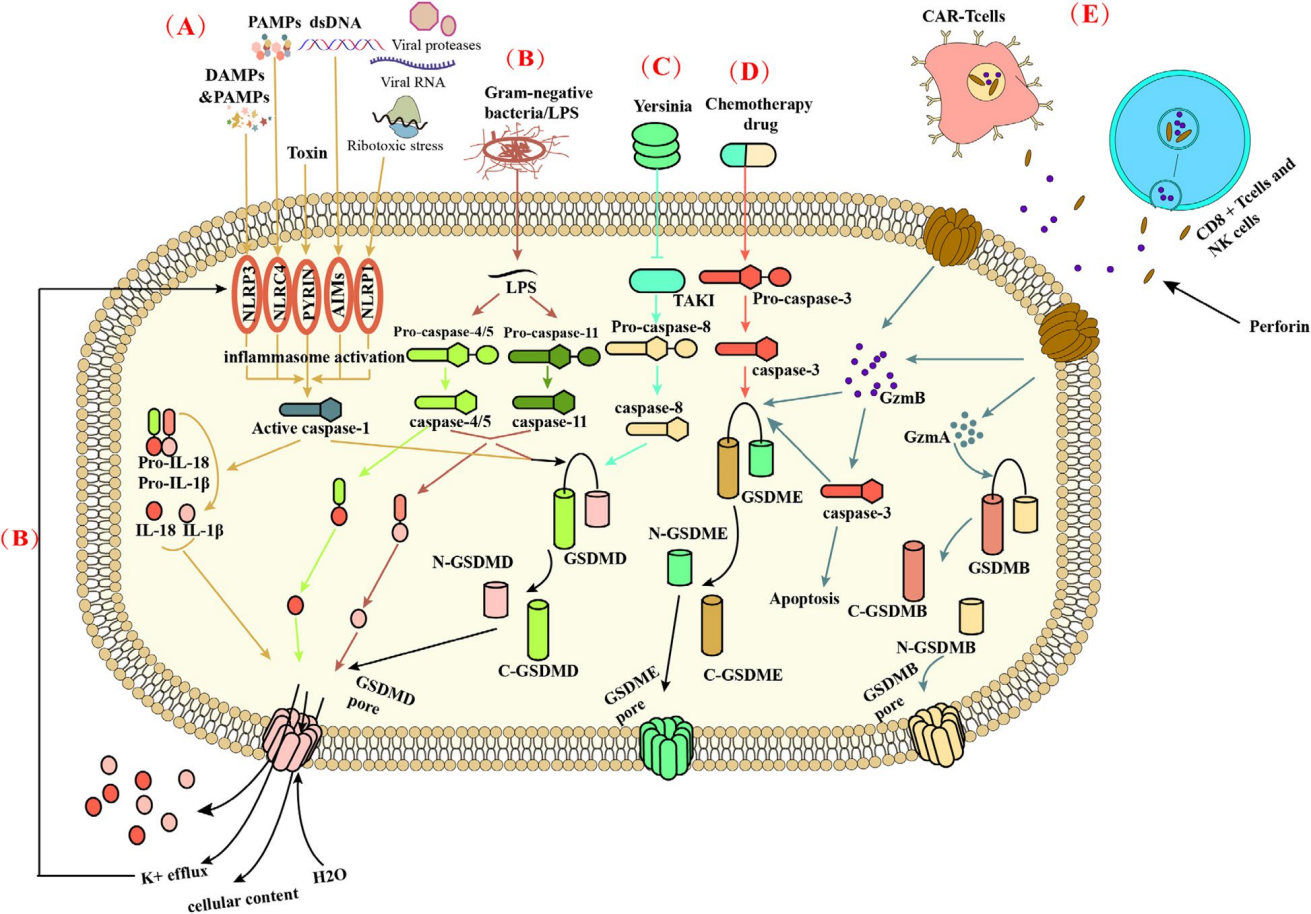
Molecular mechanisms of Pyroptosis. **A **In the canonical pathway, PAMPs and DAMPs Stimulate the corresponding inflammasome, leading to the activation of the inflammasome. Activated inflammasome can lead to the activation of caspase-1. Then, caspase-1 cleaves GSDMD form the N-terminus and C-terminus of the GSDMD. The N-terminus of GSDMD perforates the cell membrane by forming nonselective pores in the cell membrane, thus causing water influx, lysis, and death. At the same time, activated caspase-1 can promote the conversion of pro-IL-1β and pro-IL-18 to IL-1β and IL-18, which are out of the cells from the previously formed pores. **B **In the noncanonical pathway, cytosolic LPS activates caspase-4/5 and caspase-11, leading to pyroptosis through GSDMD cleavage. GSDMD-NT can also create pores in the cell membrane, causing K + and cellular content efflux, cell swelling, and rupture. This process facilitates the maturation and release of pro-IL-18 and IL-Iβ through K + efflux. Recent studies have revealed that activated human caspase-4/5 can directly cleave and activate IL-18, unlike mouse caspase-11. Additionally, all caspase-4/5/11 can cleave IL-1β to inhibit IL-1β signaling. **C **At Yersinia infection, the effector protein (Yopj) expressed by Yersinia can promote the conversion of pro-caspase-8 to caspase-8, thus mediating the pyroptosis caused by GSDMD. **D **Chemotherapy drugs can allow pro-caspase-3 to convert to caspase-3. And then caspase-3 cleaves the GSDME, resulting in pyroptosis. **E **In the granzyme-mediated pathway, CAR-T cells, CD8^+^T cells, and NK cells rapidly activate caspase-3 in target cells by releasing GzmB, and then GSDME was activated, causing extensive pyroptosis and apoptosis. At the same time, GzmA released from CD8^+^T cell and NK cell could promote cleavage of GSDMB into the N and C terminal. The N terminal of GSDMB forms holes in the cell membrane leading to pyroptosis

Canonical pyroptosis, originating from inflammasome activation, involves the activation of caspase-1, the cleavage of GSDMD, and the subsequent release of IL-1β and IL-18 [23]. (Fig. 2) The assembly of inflammasomes begins with germline-encoded pattern recognition receptors (PRRs) that recognize pathogen-associated molecular patterns (PAMPs), danger-associated molecular patterns (DAMPs) and homeostasis-altering molecular processes (HAMPs) [23–26].

Following the combination of PRRs with the stimuli, the apoptosis-associated speck-like protein (ASC) is activated [23, 24, 26]. Subsequently, ASC oligomerizes and uses its caspase activation and recruitment domain (CARD) to bind to the CARD of pro-caspase [23, 24, 26]. In general, the canonical inflammasome, a large multiprotein complex, is assembled with an activated PRR, ASC, and pro-caspase-1 [23]. Inflammasome activation can activate caspase-1, leading to the maturation and secretion of IL-1β and IL-18 [27]. Alternatively, Caspase-1 activation can cleave the pore-forming protein GSDMD to generate GSDMD N-terminal domain (GSDMD-NT), which then oligomerizes to create large pores in membranes, resulting in membrane rupture and cell death [23].

Notably, Nucleotide-binding and oligomerization domain-like receptors (NLRs), belonging to cytoplasmic PRRs, are crucial members of four major subfamilies of PRRs [28]. NLRs include the NOD, NLRP, and IPAF subfamilies [29]. The most prominent inflammasome-forming members in the NLR family are NLRP1, NLRC4, AIM2, PYRIN and NLRP3 [30]. While most inflammasomes are assembled with three components, exceptions exist. For example, NLRP1 and NLRC4 interact directly with pro-caspase-1 without the need for an ASC adaptor [31–33]. Additionally, recent research has identified the CARD8 inflammasome as part of the canonical pathway [34]. There are at least six classical pathway inflammasomes, each with specific activators [26]. Human NLRP1 inflammasomes respond to viral proteases, ribotoxic stress, and viral RNA [35]. NLRC4 inflammasomes are activated by PAMPs like flagellin, T3SS needle and rod proteins [36]. AIM2 inflammasomes respond to double-stranded DNA [37, 38]. PYRIN inflammasomes are triggered by pathogenic toxins, such as cytotoxic TcdB [39]. CARD8 recognizes specific triggers like HIV-1 protease and DPP8/9 inhibitor [34, 40]. The well-studied NLRP3 inflammasome can be activated by various stimuli, including nucleic acids, bacterial pore-forming toxins, and crystalline structures like monosodium urate and cholesterol [41–43]. Activation of the NLRP3 inflammasome occurs through two steps: activation of K^+^/Ca^2+^ outflow and mitochondrial and lysosomal-related damage.

In conclusion, the canonical pathway is crucial for host defenses by facilitating the release of inflammatory components like interleukins and DAMPs through inflammasome-induced pyroptotic cell death [2].

### The non-canonical pyroptosis pathway

The non-canonical pathway of pyroptosis, unlike the canonical pathway initiated by PRRs recognizing PAMPs and DAMPs, is specifically triggered by caspase-4 and-5 in humans, and caspase-11 in mice, to directly detect lipopolysaccharide (LPS), a component of the outer membrane of gram-negative bacteria [44]. (Fig. 2) This distinctive characteristic highlights the specialized immune response aimed at combating gram-negative bacterial infections.

Shi et al. revealed that murine caspase-11 and human caspase-4 and-5 could directly detect LPS without requiring an upstream signaling cascade. Upon interaction with LPS, procaspase-11 undergoes oligomerization, leading to activation of caspase-11 [14]. The CARD domain of caspase-11 interacts with LPS to form a the procaspase-11-LPS complex known as the non-canonical inflammasome [44]. Non-canonical inflammasome sensors have a unique ability to recognize intracellular bacteria and LPS, activating caspase-4, caspase-5 (in humans), or caspase-11 (in mice) [45, 46]. GSDMD, a ubiquitous substrate shared by both canonical and non-canonical pathways, undergoes cleavage by activated caspases specifically targeting the hinge region situated between its N-terminal and C-terminal domains [45, 46]. The C-terminal domain serves to regulate and constrain the activity of the N-terminal domain, preventing it from exerting its cytotoxic effects. On the other hand, the N-terminal domain, when released from the autoinhibition of the C-terminal domain through cleavage, demonstrates cytotoxic activity. This cleavage process is crucial in activating the cytotoxic potential of GSDMD, allowing it to perform its role in cellular processes [45, 46]. Active GSDMD-NT then interacts with acidic phospholipids on the plasma membrane, forming oligomeric death-inducing pores that increase intracellular osmolality, leading to cytolysis and pyroptosis [46].

Previous studies have demonstrated that Caspase-4/5/11 does not directly cleave pro-IL-18 and pro-IL-Iβ. Instead, they facilitate the maturation and release of pro-IL-18 and IL-Iβ by K^+^ efflux through GSDMD-NT pores. This efflux of K^+^ triggers the formation of the NLRP3 inflammasome and activation of caspase-1, ultimately leading to pyroptosis [47]. Upon stimulation by LPS, activated caspase-11 cleaves pannexin-1 cleavage, causing ATP release, K^+^ efflux and subsequent activation of NLRP3/caspase-1 by ion channel purinergic 2X7 receptor (P2X7R) [48]. Recent studies have shown that activated human caspase-4/5, unlike mouse caspase-11, can directly cleave and activate IL-18 [49–51]. Caspase-4 cleaves pro-IL-18 at the same tetrapeptide site and efficiency as caspase-1 [49, 50]. While all caspase-4/5/11 cleave IL-1β to inhibit IL-1β signaling. In summary, noncanonical inflammasomes offer an alternative pathway for IL release that does not require canonical inflammasomes or caspase-1 [51].

### Other caspase-induced pyroptosis

In addition to cell pyroptosis in both pathways described above, caspase-3 and caspase-8 also play a critical role in cell pyroptosis. Recent research in 2017 revealed that GSDME can convert caspase-3-mediated apoptosis induced by TNF or chemotherapy drugs to pyroptosis, providing new insights into cancer chemotherapy [16]. It was found that high levels of GSDME promote rapid pyroptosis following caspase-3 activation, while low levels promote apoptosis [52]. Rogers et al. demonstrated that the mitochondrial pore formed by GSDME-NT can release cytochrome C, activate apoptotic bodies, and positively regulate the cleavage of Caspase-3 and GSDME, further promoting pyroptosis [53]. These studies support the use of pyroptosis in clinical tumor treatment as a strategy to overcome apoptosis resistance in cancer therapy. However, GSDME is often silenced in most cancer cells and expressed in many normal tissues [54–56]. Therefore, GSDME-mediated pyroptosis contributes to toxic side effects observed during cancer chemotherapy. (Fig. 2).

In mouse macrophages, the effector protein YopJ expressed during Yersinia infection, has been found to inhibit TGF-β-activated kinase 1 (TAK1) and induce caspase-8 mediated cleavage of GSDMD [57]. Additionally, upon tumor necrosis factor-α (TNF-α) stimulation, Caspase-8 specifically cleaves GSDMC to produce the GSDMC N-terminal domain, resulting in the formation of membrane pores and triggering pyroptosis [58].

In addition to caspase-3 and caspase-8, certain apoptotic caspases play distinct roles in pyroptosis. Caspase-6 is involved in regulating GSDMD processing through Z-DNA binding protein 1 (ZBP1)-mediated inflammasome activation during influenza A virus (IAV) infection [59]. Caspase-7, on the other hand, can inhibit pyroptosis by cleaving GSDMB [60]. In summarize, apoptotic caspases can either directly act on pyroptosis (caspase-3/7/8) or indirectly affect pyroptosis by affecting other substances (caspase-6).

### The granzyme-induced pyroptosis

In addition to intracellular caspase enzymes, certain enzymes secreted by cells in the microenvironment, such as immune cells, can trigger pyroptosis through paracrine pathways. Cytotoxic T lymphocytes (CTLs) and NK cells release serine protease granzymes into target cells using perforin, ultimately leading to cell death in cellular immunity [20, 61]. A 2020 study revealed that GzmA secreted by cytotoxic lymphocytes cleaves GSDMB, particularly at Lys244 within the interdomain linker [20]. The delivery of GzmA into GSDMB-reconstituted cells via electroporation or perforin to induce extensive pyroptosis was hindered by the K229A/K244A mutation of GSDMB and suppression of GzmA expression. Additionally, Interferon-γ (IFN-γ) was found to enhances GzmA-induced pyroptosis by increasing the expression of GSDMB [20].

In a 2020 study, GzmB was found to GzmB can induce pyroptosis by cleaving GSDME, leading to the conversion of noninflammatory apoptosis to pyroptosis in GSDME-expressing cells [19]. GzmB present in NK cells triggers caspase-independent pyroptosis in target cells by directly cleaving GSDME at the same site as caspase-3 [19]. Additionally, GzmB can indirectly promote GSDME-dependent cell pyroptosis through the activation of Caspase-3 [19].

CAR-T therapy is an immunotherapy that targets cancer cells by altering the patient's immune system [62]. Studies indicate that CAR-T cells prompt caspase-3 activation in target cells by releasing GzmB, leading to GSDME cleavage and subsequent pyroptosis [63]. This pyroptosis-induced process triggers caspase-1 activation in macrophages, resulting in cytokine release and cytokine release syndrome (CRS) [64, 65]. Overall, the GzmA-GSDMB and GzmB-GSDME pathways play a crucial role in the body's antitumor immune response, offering novel insights for tumor immunotherapy.

## Time on tumor pyroptosis

The success of tumor immunotherapy may be influenced by the heterogeneity of the tumor immune microenvironment and its components [66, 67]. Recent research suggests that harnessing pyroptosis to modulate TME could enhance antitumor effects. Understanding how the TIME and its components affects pyroptosis in tumor cells may help address the variability in patient responses to immunotherapy. Herein, we focus on the role and therapeutic potential of various immune cells within TIME in relation to pyroptosis of the tumor cells.

### T cells

**Fig. 3:**
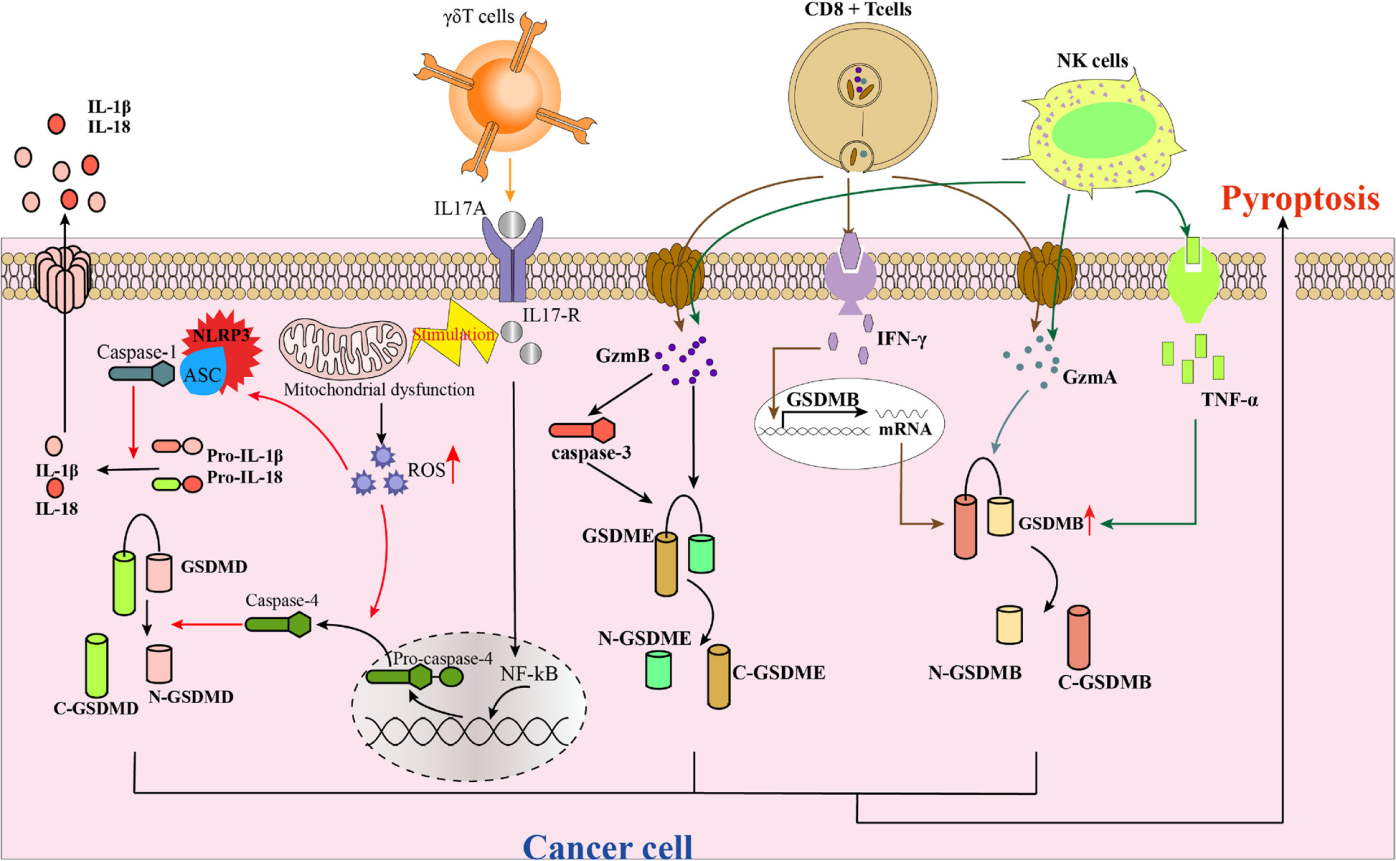
Molecular mechanisms of T cells and NK cells on pyroptosis. CD8^+^T cells and NK cells induce cancer cell pyroptosis by secreting GzmA and GzmB, which are enzymes capable of cleaving GSDMB and GSDME, respectively. In addition, activated cytotoxic lymphocytes release IFN-γ, upregulating GSDMB expression in cancer cells. Activated NK cells release TNF-α, increasing the expression of endogenous GSDMB and promoting pyroptosis induced by GzmA. IL-17A, a pro-inflammatory cytokine, mainly secreted by γδT cells in the immune microenvironment of colorectal tumours, could promote intracellular ROS accumulation by inducing mitochondrial dysfunction. Furthermore, IL-17A could induce pyroptosis of colorectal cancer cells and significantly upregulate the secretion of inflammatory factors

Different subtypes of T cells in the TIME have distinct roles in inducing or influencing tumor cell pyroptosis. Cytotoxic CD8 + T cells are the main executors of transformed and cancer cells in cancer immunotherapy through the granzyme pathway, leading to tumor cell pyroptosis [68]. The release of GzmA/B by CTLs into tumor cells via perforin directly or indirectly triggers GSDM-dependent pyroptosis, resulting in immune activation. IFN-γ plays a crucial role in the process of tumor pyroptosis. Activated cytotoxic lymphocytes release IFN-γ, which upregulates GSDMB expression, thereby promoting pyroptosis in esophageal carcinoma cell lines (OE19 and OE33) and a breast cancer cell line (HCC1954), thereby enhancing the tumor cell pyroptosis induced by T cells. Furthermore, IFN-γ significantly enhances pyroptosis in HCC1954 cells and SW837 cells through the cytosolic delivery of GzmA [20] (Fig. 3).

A study has shown that Interleukin-17A (IL-17A), a pro-inflammatory cytokine primarily secreted by γδT cells in the immune microenvironment of colorectal tumors, can enhance intracellular reactive oxygen species (ROS) accumulation by disrupting mitochondrial function [69]. Additionally, IL-17A can trigger pyroptosis in colorectal cancer cells and increase the release of inflammatory factors [69]. The mechanism by which IL-17A induces mitochondrial dysfunction and ROS accumulation may involve altering the expression levels of components in the mitochondrial respiratory machinery, impairing mitochondrial respiratory capacity and leading to elevated ROS levels [69, 70]. Interactions between the IL-17 family andtheir receptors activate nuclear factor kappa-B (NF-κB) signaling, resulting in the secretion of various pro-inflammatory mediators [71, 72]. This activation also increases the expression of active forms of Caspase-4, caspase-1, and GSDM, along with the upregulation of NLRP3, IL-1β, and IL-18 [69]. In summary, activated mitochondrial dysfunction and caspase-1 and caspase-4 are involved in mediatingIL-17A-induced Pyroptosis [69].

### NK cells

NK cells are effector cells of the innate immune system that play a crucial role in controllingintracellular pathogens and tumors [73]. Increased expression of GSDME in cancer cells not only boosts NK cell numbers but also enhances their ability to kill tumor cells [19]. This process leads to the production of various factors, such as reactive nitrogen species, ROS, reactive aldehydes, cytokines, chemokines, and growth factors [74]. The activation of TME by NK cells shifts it towards an immunostimulatory state. Furthermore, NK cell-induced tumor cell pyroptosis effectively triggers the release of intracellular pro-inflammatory factors, rapidly initiating tumor inflammation [74].

NK cells play a crucial role in inducing pyroptosis through GSDME [75] (Fig. 3) A recently study showed Schisandrin B(Sch B) alone induces apoptosis in human hepatocellular carcinoma (HepG2) cells, but the presence of NK cells shifts this response towards pyroptosis. Sch B, an active ingredient from Schisandrae chinensis (Turcz.) Baill. (Schisandraceae) Fructus, has been shown to possess diverse pharmacological effects, such as antitumor, antioxidant, anti-inflammatory, and hepatoprotection [76]. The mechanism underlying the promotion of pyroptosis by NK cells in Sch B-treated HepG2 cells involves the activation of caspase-3 and GSDME [77].

In a recent study conducted in 2020, it was observed that Interferon-α (IFN-α), Interferon-β (IFN-β), IFN-γ, and TNF-α to a lesser extent, increased the expression of endogenous GSDMB and facilitated pyroptosis induced by GzmA [20]. This suggests that interferons have the potential to amplify NK cell-mediated pyroptosis. Although NK cells are known for their ability to eliminate tumor cells by inducing pyroptosis and performing immune functions, these functions are often suppressed. Within the TME, cancer-associated fibroblasts and myeloid-derived suppressor cells have been implicated in the inhibition of NK cell activation through various mechanisms [78, 79].

CAR‐NK cells combine the targeted specificity of antigens with the subsequent intracellular signaling ability of the receptors to enhance their anticancer functions [80]. Indeed, CAR‐NK cells can be adapted to recognize various antigens, hold higher proliferation capacity, and in vivo persistence, show improved infiltration into the tumors, and have the ability to overcome the resistant tumor microenvironment leading to sustained cytotoxicity against tumors [80]. The risk of on‐target/off‐tumor toxicity to normal tissues is relatively low owing to the limited lifespan of CAR‐NK cells in circulation [81].

### Tumor associated macrophages

**Fig. 4:**
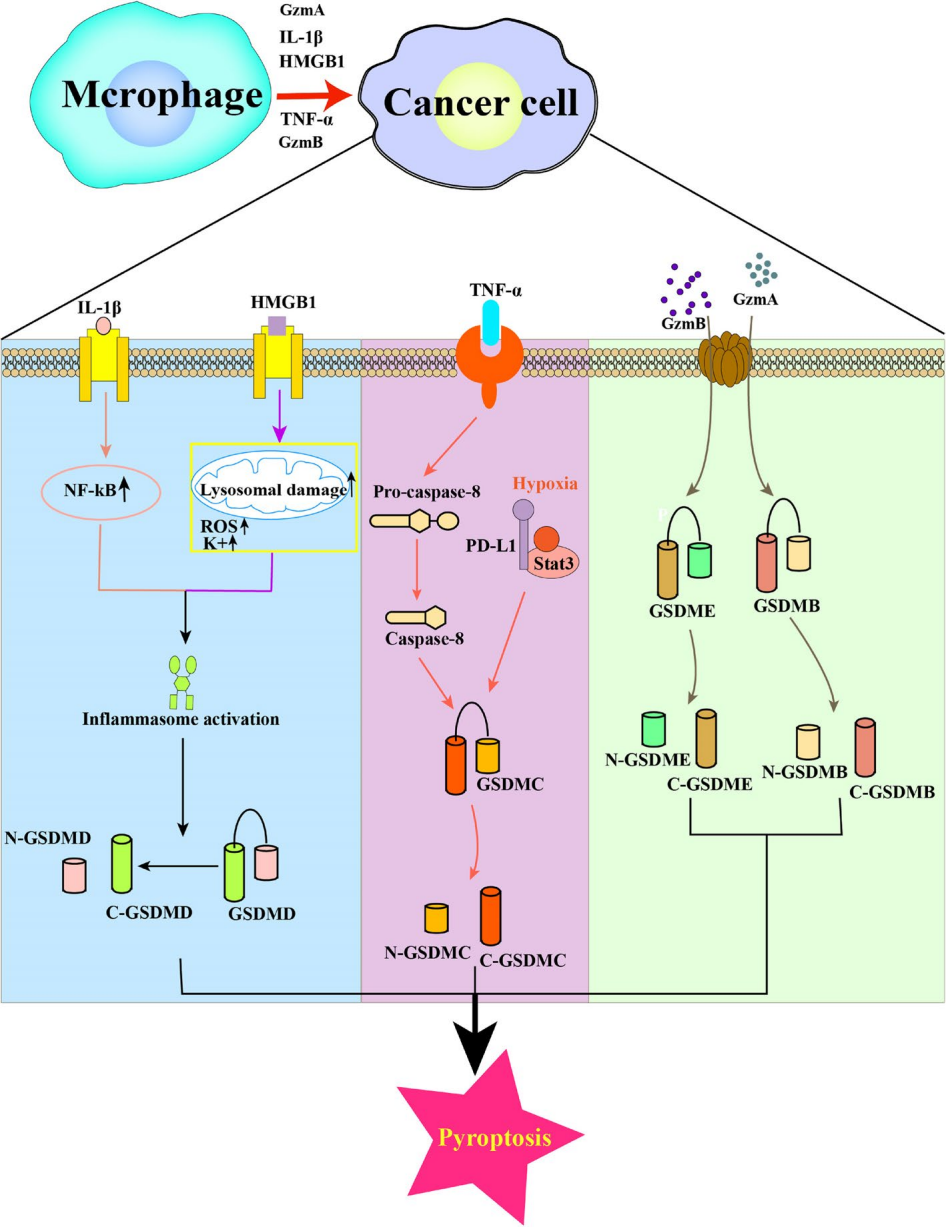
Molecular mechanisms of TAMs on pyroptosis Macrophages can regulate the activation of inflammatory vesicles by secreting cytokine IL-βto activate NF-kB or secreting the pro-inflammatory mediator HMGB1, which promotes the pyroptosis process. TAMs can also affect the caspase-8 cleavage of GSDME by secreting TNF-α. The hypoxia-induced formation of the nPD-L1/p-Stat3 complex increases the expression of GSDMC, cleaved by TNFα-activated caspase-8 to mediate pyroptosis. Besides, TAMs releases pro-inflammatory cytokines to trigger CRS to be involved in the granzyme pathway, which is relevant in CAR-T cell therapy

TAMs play crucial roles in tumorigenesis, development, and metastasis. They consist of M2 and small populations of M1 cells, which exhibit cytotoxic effects on tumor cells and interact with pyroptosis signaling pathways [82, 83]. The classification of macrophages is complex, with two main types based on metabolic functions. M1 macrophages have anti-inflammatory and anti-tumor properties due to increased secretion of inflammatory cytokines [84]. M2 macrophages are involved in collagen fiber repair, produce immunosuppressive cytokines, and contribute to processes such as angiogenesis, tissue repair, immunosuppression, and tumor promotion [84]. In the tumor environment, TAMs play dual roles as both tumor promoters and immune suppressors by initiating tumor growth and modulating the immunosuppressive tumor microenvironment through various mechanisms, including the expression of cell surface receptors and secretion of cytokines, chemokines, and enzymes [85]. TAMs are also implicated in pyroptosis through both canonical and non-canonical pathways, as well as through the granzyme action of GSDMB [82] (Fig. 4).

Macrophages can regulate the activation of inflammatory vesicles through two processes involving caspase cleavage by GSDMD [82]. In the first process, TAMs secrete cytokine IL-β to activate NF-kB, which in turn regulates various genes that promote tumor growth, migration, angiogenesis, and pro-apoptotic or pyroptosis through various pathways [86, 87]. In the second process, TAMs respond to various pathogenic factors by secreting pro-inflammatory mediator HMGB1 when cells are damaged and DAMPs are released [88]. HMGB1, a typical DAMP, plays a role in the activation of inflammatory vesicle in the TME. Additionally, TAMs can enhance the activation of inflammatory vesicle by accelerating ATP release, which acts on P2X7R on the cell surface, opens cell membrane cation channels, and reduces intracellular K^+^ level [89]. These findings suggest that TAMs secrete cytokines to activate the inflammatory vesicles and promote pyroptosis.

TAMs can impact caspase-8 cleavage of GSDME. Hou and his colleagues have demonstrated that macrophages, acting as a significant source of tumor necrosis TNF-α within TME, can activate caspase-8 by secreting TNF-α, thereby contributing to pyroptosis [82]. Specifically, under hypoxic conditions, formation of the nPD-L1/p-Stat3 complex leads to increased expression of gasdermin C (GSDMC), which is cleaved by TNF-α-activated caspase-8 to induce pyroptosis. This process results in tumor necrosis in hypoxic regions, ultimately promoting tumor progression and inhibiting antitumor immune response [90]. Furthermore, studies suggest that TAMs release pro-inflammatory cytokines to trigger CRS and participate in the granzyme pathway, which is relevant for CAR-T cell therapy [63] (Fig. 4).

### Tumor-associated neutrophils

TANs are formed by the recruitment of neutrophils to tumors [91]. After recruitment to the TME, neutrophils release cytokines and enzyme-like substances that affect the recruitment and activation of inflammatory cells [92]. The role of neutrophils in tumor biology, such as tumor progression and invasion, is a topic of debate. Numerous studies have suggested that neutrophils play a tumor-promoting role in cancer progression [93, 94]. On the contrary, several other studies have shown that neutrophils can exert antitumor effects by activating the immune response against tumors and promoting the clearance of tumor cells [95].

**Fig. 5:**
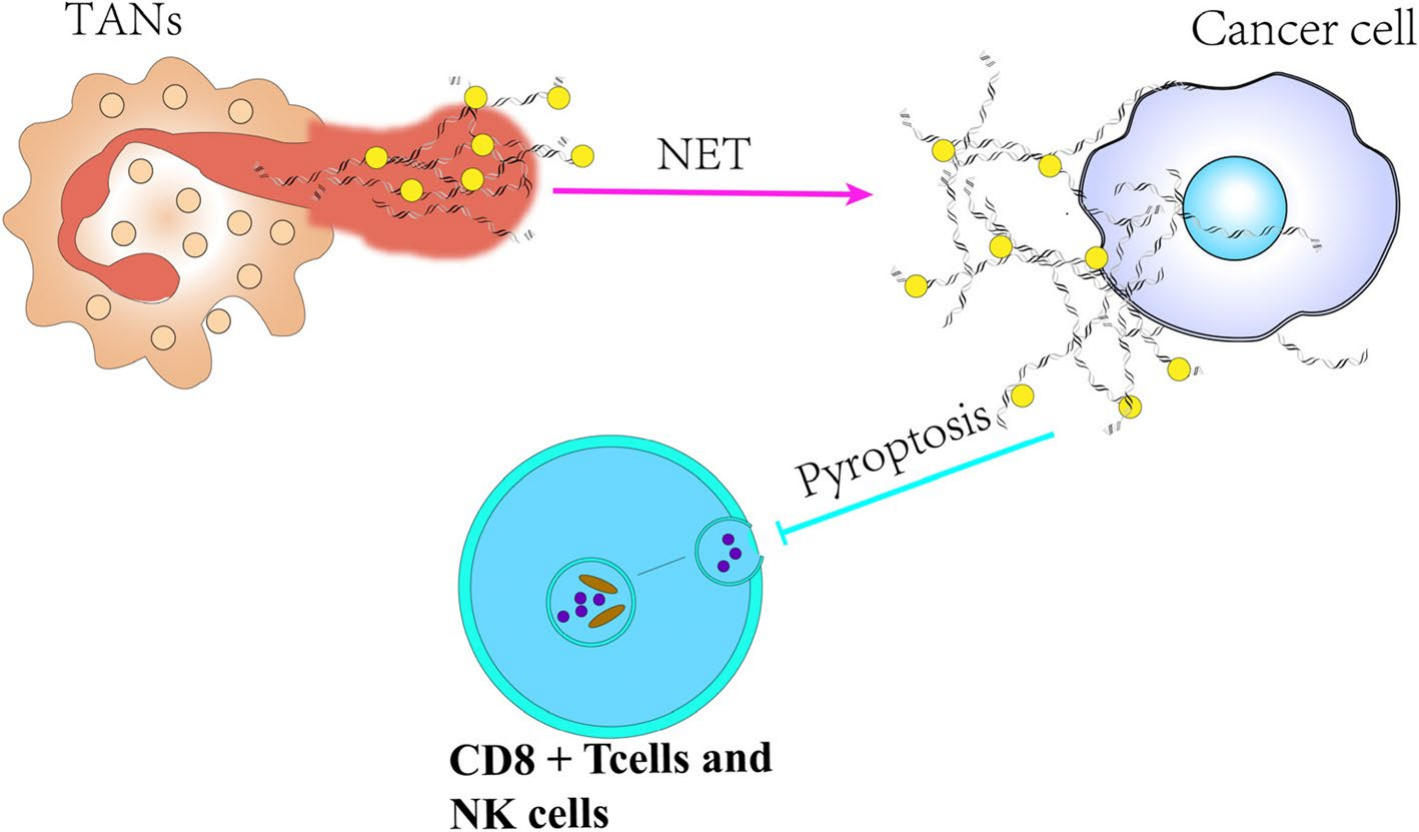
Molecular mechanisms of tumor-associated neutrophils in inhibiting pyroptosis. Neutrophils are stimulated which can release Nets. Then, NETs form a barrier on the surface of cancer cells thereby impeding the ability of CD8^+^T cells and NK cells to induce pyroptosis in tumor cells

In 2004, Brinkmann et al. observed a unique form of neutrophil degranulation characterized by DNA fibers adorned with granule proteins, initially referred to as neutrophil extracellular traps (NETs) [96]. Fuchs et al. explained that upon activation, neutrophils become highly phagocytic and undergo morphological changes that culminate in NETs formation [97]. NETs are extracellular strands of decondensed DNA fibers bound to histones and various neutrophil granule proteins, such as matrix metalloproteinase (MMP), neutrophil elastase (NE), myeloperoxidase (MPO), cathepsin G, complement factors, and other enzymatically active proteases and peptides [98]. Teijeira et al. demonstrated that NETs induced by C-X-C chemokine receptor 1 (CXCR1) and C-X-C chemokine receptor 2 (CXCR2) agonists could encase cancer cells, providing them protection from clearance and the cytotoxic effects of cytolytic CTLs and NK cells [99]. Specifically, NETs can impede the ability of CD8^+^ T and NK cells to trigger tumor pyroptosis within a certain range. Furthermore, NE present in NETs can enhance invasion and metastasis of oral squamous cell carcinoma (OSCC) by suppressing NLRP3-induced pyroptosis [100] (Fig. 5).

Importantly, NETs have been shown to play a role in the development of arterial, venous, and cancer-related thrombosis [101, 102]. These results indicate that targeting NETs could be a potentially effective strategy for decreasing thrombosis and hindering tumor advancement and metastasis.

## Immune therapy based on tumor pyroptosis

While the immune system can eliminate tumor cells through the cancer-immune cycle, tumors often develop immune evasion by shaping an immunosuppressive microenvironment [103]. Reshaping TIME to restore the tumor-killing ability of anti-tumor immune cells and form an immune-supportive microenvironment has emerged as a critical focus in tumor immunotherapy [63]. Despite advancements in therapies such as CAR-T cell therapy and ICIs, there are still limitations in the treatment and prognosis of cancer patients due to tumor immune escape mechanisms.

### CAR-T cell therapy

CAR-T cell therapy, a form of immunotherapy, involves genetically engineering T cells to target and kill tumors using antibody-derived CARs [104]. These modified T cells target inhibitory signaling molecules present in tumor cells [105]. Upon recognition of tumor-associated antigens by CARs, CAR-T cell activity is significantly increased. The process by which CAR-T cells induce tumor cell death through pyroptosis has been well documented in the granzyme pathway. Despite being a groundbreaking advancement in cancer treatment, CAR-T cell therapy encounters challenges related to efficacy, toxicity, side effects, etc. [106].

CAR-T therapy, a highly personalized form of immunotherapy, holds great promise for tumor treatment. It is characterized by superior cytotoxicity, persistence, and antigen recognition capabilities despite tumor-induced immunosuppressive influences [107, 108]. This therapy has demonstrated long-lasting antitumor immune responses in B-cell malignancies such as acute lymphoblastic leukemia, chronic lymphocytic leukemia, and non-Hodgkin’s lymphoma [109]. The positive outcomes of CAR-T therapy led to the US Food and Drug Administration (FDA) approval of anti-CD19 CAR-T cell therapy for B cell malignancies, marking a historic and unprecedented milestone [110].

Although CAR-T cell therapy play a non-negligible role in tumor treatment, various challenges hinder its therapeutic efficacy in both solid tumors and hematological malignancies. Further investigations are needed to address the toxicity and side effects associated with this therapy [106]. Major limitations include life-threatening toxicities, limited efficacy against solid tumors, resistance to B cell malignancies, antigen escape, limited persistence, poor trafficking, tumor infiltration, and as well as the presence of an immunosuppressive microenvironment [106]. Factors contributing to these limitations in solid tumor treatment include physical barriers hindering CAR-T cell entry, migration hindrance, recruitment of immunosuppressive cells, and shaping of an immunosuppressive environment [104]. CRS is a common immune-mediated toxicity characterized by fever, hypotension, and respiratory insufficiency due to elevated serum cytokine levels. Strategies such as knocking out GSDME, depleting macrophages, or inhibiting caspase-1 in mouse models have shown promise in mitigating CRS [63]. Research indicates CRS severity is correlated with GSDME and lactate dehydrogenase levels [63]. Therefore, it is crucial to not only consider the effect of CAR-T treatment but also monitor and manage the occurrence of CRS.

In order to advance therapeutic interventions, particularly in reducing drug resistance and minimizing toxic side effects, current research suggests that enhancing the efficacy of CAR-T anti-cancer therapy can be achieved through the choice of T-cell subpopulations and the modification of their nature [111]. This includes adjusting the ratio of helper T cells (CD4^+^T cells) to CD8^+^T cells in a patient-specific manner and modifying the differentiation status of T-cell modification [112]. To address potential toxic side effects, it is important to increase the selectivity of the isoform of the target antigen isoform to prevent CAR-T cells from attacking healthy tissue [111]. Although various strategies have been proposed to overcome the limitations of CAR-T therapy, many have not progressed to clinical trials. Therefore, further investigation into existing methodological approaches and the development of innovative strategies are essential to enhance anti-tumor activity and reduce toxicity.

### Immune checkpoint inhibitors

ICIs, a prominent form of immunotherapy, have received significant attention as compelling treatment options [113]. They have emerged as potent therapeutic options for a wide array of solid tumors. Among immune checkpoint regulators, CTLA-4, PD-1, and PD-L1 are prominent, drawing substantial interest in the field of oncology as promising and powerful targets for cancer therapeutics [114]. As commonly understood, cancer cells utilize various mechanisms to evade the human immune system, including evading recognition by immune cells, enhancing resistance to apoptotic pathways, and creating immunosuppressive conditions [114]. Additionally, immune checkpoints are recognized as negative regulators of immune response and play a crucial role in preventing excessive peripheral tissue damage [115]. For example, the PD-1/PD-L1 system actively suppresses T lymphocyte proliferation, cytokine production, and cytotoxicity in cancer cells, leading to fatigue and apoptosis of tumor-specific T cells, allowing cancer cells to evade immune responses [116]. While the specific mechanisms of CTLA-4 activity remain unknown, it is postulated that its presence on the surface of T cells dampens T cell activation. This occurs through the active conveyance of inhibitory signals to T cells, achieved by outcompeting CD28 in binding CD80 and CD86 [117]. Targeting PD-1, PD-L1, or CTLA-4 effectively reverses the suppression of cytotoxic T lymphocytes, leading to the elimination of tumor cells by restoring T cell functionality. In immune-competent hosts, tumors evade immune surveillance during tumorigenesis. Blocking PD-1/PD-L1 enables T cells to enhance their growth, cytotoxicity, and infiltration into tumors, ultimately reducing tumorigenesis [118]. Currently, many drugs have been developed based on studies of ICIs, such as Ipilimumab Pembrolizumab, and Atezolizumab.

In 2011, Ipilimumab became the first FDA-approved ICI after successful trials in metastatic melanoma [119]. It's a human IgG1 monoclonal antibody that targets CTLA-4. Mechanistically, it blocks the interaction between CTLA-4 and CD86/CD80 on T cells or antigen-presenting cells [120]. This interference prevents the inhibitory signals of CTLA-4 and allows CD28 to bind with CD80/CD86, ultimately promoting T-cell activation [121].

Pembrolizumab, a clinically approved PD-1 inhibitor, represents a significant advance in the treatment of unresectable, advanced, and metastatic cancer. Its FDA approval marks an important milestone in improving in the treatment outcomes for these complex cancer [122, 123]. Pembrolizumab is known for its strong binding to PD-1 with low affinity for Fc receptors and complement [124]. Pembrolizumab has become the first immune checkpoint inhibitor approved for first-line treatment in several melanomas. By preventing the suppression and deactivation of immune cells, pembrolizumab has revolutionised melanoma treatment and offers a new approach to this challenging cancer [125]. Pembrolizumab has shown significant potential in clinical trials, particularly in patients with higher levels of PD-L1, and has been approved for the treatment of multiple cancers.

Atezolizumab, a human IgG1 monoclonal antibody, has the distinction of being the first FDA-approved PD-L1 inhibitor approved by the FDA. It is used in the treatment of various cancers,including urothelial carcinoma, triple negative breast cancer, non-small cell lung cancer, and small cell lung cancer [126]. Atezolizumab is a genetically engineered PD-L1 inhibitor with a modified Fc domain designed to reduce interactions with Fcγ receptors to decrease reducing traditional antibody-dependent cell-mediated cytotoxicity. This modification is intended to enhance the drug's therapeutic efficacy of the drug while minimising potential side effects related to immune system activation [127]. This Fc domain modification has been linked to the prevention of PD-L1 expression on immune cells, resulting in a more effective anti-tumor immune response [128].

While individual ICIs have shown efficacy in the fight against, there is a growing focus in clinical practice on using combination therapies to increase their pharmacological impact and reduce potential side effects. An example of this is the apexample of this umab not only as a stand-alone treatment but also in combination with Nivolumab, a PD-1 inhibitor [120]. This co-administration therapy has been approved for the treatment of unresectable (advanced) melanoma, renal cell carcinoma, and colorectal cancer with either high microsatellite instability (MSI-H) or mismatch repair-deficient (dMMR) status [120]. The simultaneous use of these ICIs is intended to synergistically improve the therapeutic response, and represents a significant advance in cancer treatment [120].

### Enhancing pyroptosis in tumor immunotherapy

Although current tumor immunotherapy has shown significant success, it still faces challenges in achieving efficacy in most patients [129]. Take ICIs for example, many tumours respond poorly or not at all to ICIs, in part due to a lack of tumour-infiltrating lymphocytes (TILs) [130]. As a result, there is a pressing need for additional strategies to enhance antitumor immunity, such as converting these immunologically “cold” tumors into “hot” tumors [130]. In contrast to apoptosis, which tumor cells often resist, numerous studies suggest that harnessing pyroptosis in the tumor microenvironment can trigger a robust immune response, potentially offering more effective cancer therapy options and improving patient survival [129, 131, 132].

Pyroptosis is closely related to the immune system. On the one hand, pyroptosis can stimulate the immune system through by activating immune cells and immune factors [133]. Pyroptosis-produced cytokines can attract immune cells and ignite the immune system, potentially improving the efficacy of tumor immunotherapies [134]. On the other hand, immune cells like T cells and NK cells in the TIME can induce pyroptosis in tumor cells by releasing perforin and granzyme. Various therapeutic approaches can boost the immune system by inducing pyroptosis directly or indirectly [135]. Combining pyroptosis induction with ICIs has shown synergistic antitumor effects, even in ICI-resistant tumors [75]. However, inducing pyroptosis alone may not effectively inhibit tumors, highlighting the importance of combining pyroptosis inducers for cold tumors [75]. In CAR-T Cell Therapy, CAR-T cells induce pyroptosis in tumor cells by activating the caspase-3/GSDME pathway through GzmB release [19]. Nevertheless, pyroptosis is also linked to the toxicity and side effects of this therapy. Therefore, it is important to further investigate the role of pyroptosis in immunotherapy to optimize treatment efficacy and minimize associated toxicities and side effects.

## Conclusions

Although considerable progress has been made in understanding the molecular mechanism of pyroptosis through intensive research, further investigations are needed to explore the signalling pathway, additional regulatory factors, functions of other GSDM family members, and the pathological implications of pyroptosis. The interaction between pyroptosis and tumors is intricate and multifaceted. On the one hand, pyroptosis inhibits tumor cell proliferation, invasion, and metastasis [136]. On the other hand, pyroptosis shapes an immunosuppressive microenvironment suitable for tumor cell growth to promote tumor growth. Moreover, the specific regulatory mechanisms of pyroptosis in different types of tumors and stages of tumor development remain unclear due to the complex nature of these relationships [137]. Furthermore, the regulation of tumor cell pyroptosis by various immune cells is complex and varies depending on the distribution of immune cells and subtypes within a specific tumor. This complexity highlights the need for in-depth studies to unravel the regulatory mechanisms of pyroptosis in specific tumors. Overall, tumor immunotherapy encounters numerous challenges.

In recent years, tumor immunotherapy has seen significant advance, particularly in CAR-T therapy and tumor ICIs. However, challenges such as the instability of CAR-T efficacy, toxic side effects, and tolerance issues hindered their its clinical progress. It is imperative to investigate existing strategies and develop innovative approaches to improve antitumor efficacy and minimize toxicity. Research on immune checkpoint inhibitors has also faced obstacles, despite recent achievements. The pace of research in this area has slowed, and the decrease in experimental patients poses a significant challenge to clinical trials involving ICIs.

Targeting pyroptosis as a novel therapeutic strategy for the development of anticancer drugs destined for clinical use is an intricate and labor-intensive journey. Crafting potent medications that precisely activate cell pyroptosis in human systems, while simultaneously adhering to rigorous safety testing protocols, continues to pose a formidable challenge within the realm of pharmaceutical research [75]. The integration of targeted therapies, whether as inducers or inhibitors of pyroptosis, with immunotherapy modalities holds immense promise in this endeavor. This multifaceted approach has the potential to unlock new frontiers in cancer treatment, providing patients with more effective and personalized therapeutic options [129]. Additionally, the synergistic benefit of combining chemotherapy and ICIs in cancer therapy has been widely reported, but the role of pyroptosis in chemotherapy toxicity requires further investigation [129]. Moreover, the DNA damage inflicted by radiotherapy can provoke cell pyroptosis via diverse signaling pathways, leading to significant antitumor efficacy when synergistically paired with immunotherapy [137–139]. This synergistic approach harnesses the power of both treatment modalities to achieve robust therapeutic outcomes. In essence, the synergistic alliance of targeted therapy, radiotherapy, and chemotherapy with immunotherapy holds immense potential in the realm of antitumor therapy. Nevertheless, the precise sequence and timing of these combined treatment modalities are pivotal considerations that significantly influence therapeutic efficacy and ultimately, patient prognosis [130].

### Supplementary Information

Supplementary Material 1.

## Data Availability

This article will be accessed on online upon publication.
