# Supplementary material for: The crosstalk between immune cells and tumor pyroptosis: advancing cancer immunotherapy strategies
Source: J Exp Clin Cancer Res. 2024 Jul 10;43:190. doi: 10.1186/s13046-024-03115-7 (PMC11234789; doi:10.1186/s13046-024-03115-7)
Supplement: Supplementary file 1 — Supplementary Material 1. [file 13046_2024_3115_MOESM1_ESM.docx]

**Dear *Journal of Experimental & Clinical Cancer Research* editorial team,**

We are very pleased to resubmit our manuscript “The Crosstalk between Immune Cells and Tumor Pyroptosis: Advancing Cancer Immunotherapy Strategies” to *Journal of Experimental & Clinical Cancer Research*. Following the suggestions of the reviewers, we have made some modifications and improvements to our manuscript.

A detailed point-by-point response is appended below and all changes in the revised manuscript are highlighted in red. We sincerely thank the editor and all reviewers for their valuable feedback that we have used to improve the quality of our manuscript.

**Point-by-point responses to the reviewers’ reports:**

**Comment 1:** Lines 35-40: This emerging type of regulated cell death significantly influences cancer modulation, antitumor immunity, and the prognosis of cancer patients(2). Pyroptosis's impacts are not only inhibiting tumor cell proliferation but also shaping an immunosuppressive microenvironment which promotes tumor growth.

**Response 1:** Thank you so much for your insightful suggestions. We have carefully revised the sentence in line 40, highlighted in red, by replacing "suitable for tumor cell growth to promote tumor growth" with "which promotes tumor growth." This change has made the text more concise and accurate. Your professional guidance is invaluable to us, and we will continue to strive for improving the quality of our work.

**Comment 2:** Line 41…… "components"..: specify what these components are.

**Response 2:** The components referenced in line 41 pertain specifically to the structural elements within the tumor microenvironment (TME), namely, extracellular matrix proteins and cytokines. Therefore, the sentence has been revised to clarify: "The tumor microenvironment (TME) encompasses a heterogeneous mix of non-tumor cells like immune cells, stromal cells, and blood vessels. Additionally, it includes structural components within the tumors, such as extracellular matrix proteins and cytokines. These diverse elements interact with the tumor cells, collectively contributing to tumor growth, progression, and response to therapeutic strategies." This clarification aims to provide a deeper understanding of the intricate nature of the TME and its role in tumor biology. These sentences is on lines 42-46 and is highlighted in red.

**Comment 3:** Line 46: natural killing: you mean natural killer (NK).

**Response 3:** I appreciate the reviewer's correction. It was indeed a spelling mistake. We intended to refer to "Natural Killer Cells" (abbreviated as NK cells), not "natural killing". This typo could potentially lead to misunderstandings for readers. We sincerely apologize for the error and have made the necessary correction in the manuscript. Specifically, in line 49, highlighted in red, we have replaced "natural killing" with "natural killer (NK) cells".

**Comment 4:** Lines 64-65: leading to the mature and release of Interleukin-1β. Do you mean maturation??

**Response 4:** Thank you once again for your careful review and valuable feedback. In this section, we have indeed made a terminological mistake. Our original intention was to convey that the process leads to the "maturation and release of Interleukin-1β". In biology and medicine, when describing the maturing process of cells or molecules, the term "maturation" is typically used, rather than "mature". Therefore, in line 69, which is highlighted in red, we replaced "mature" with "maturation" to ensure more accurate and professional language usage.

**Comment 5:** Line 112:… "The well stained NLRP3 inflammasome can be activated by various stimuli,". What do you mean by stained?

**Response 5:** Thank you for pointing out the error. It was indeed a typo, and "well stained" should be corrected to "well-studied." What we intended to convey was that the "well-studied NLRP3 inflammasome can be activated by various stimuli." We have made the necessary correction in line 123，which is highlighted in red.

**Comment 6:** Lines 135-136:" the shared substrate GSDMD is cleaved by activated caspases". Share with whom?

**Response 6:** Thank you very much for pointing out the issue in our manuscript. We recognize that we did not explicitly state which components GSDMD shares its substrate status with, which could have caused confusion for readers. To address this, we have made the necessary correction and clarification in line 142-144 of the manuscript. The revised sentence is: “GSDMD, a ubiquitous substrate shared by both canonical and non-canonical pathways, undergoes cleavage by activated caspases specifically targeting the hinge region situated between its N-terminal and C-terminal domains (45, 46).”

**Comment 7:** Lines 137-138: this sentence:" The cleavage releases an intramolecular autoinhibition exerted by the GSDMD C-terminal domain (GSDMD-CT) on the GSDMD-NT, showing the cytotoxic activity of the N-terminal domain", is unclear to me, and consequently the rest of the paragraph. I have a hard time picturing the mechanism. Explain better and/or add figure to show mechanism. A scientist who knows pyroptosis may understand, one who is not so knowledgeable like me, does not.

**Response 7:** We appreciate the reviewer's comments regarding the clarity of Lines 137-138 and the subsequent paragraphs discussing the mechanism. We recognize that the explanation may have been insufficient for those who are not as familiar with pyroptosis. To address this concern, we have made significant revisions to the content in order to enhance its readability and clarity. We made modifications in lines 145-149: “The C-terminal domain serves to regulate and constrain the activity of the N-terminal domain, preventing it from exerting its cytotoxic effects. On the other hand, the N-terminal domain, when released from the autoinhibition of the C-terminal domain through cleavage, demonstrates cytotoxic activity. This cleavage process is crucial in activating the cytotoxic potential of GSDMD, allowing it to perform its role in cellular processes.” Furthermore, we have included a figure 2B that visually illustrates the mechanism involved. This figure provides a graphical representation of the process, which we hope will make it easier for readers to visualize and understand the steps involved.

**Comment 8:** lines 248-249: "Furthermore, NK cell-induced cell pyroptosis tumor cell pyroptosis….. Sentence unclear.

**Response 8:** We apologize for the linguistic error in the sentence. It was indeed a mistake to repeat "cell pyroptosis" twice, causing confusion. To correct this issue, we have revised the sentence by removing the redundant "cell pyroptosis" to ensure clarity. The revised sentence reads: "Furthermore, NK cell-induced tumor cell pyroptosis..." in line 261-262, which is highlighted in red. This change removes the linguistic redundancy and makes the meaning of the sentence more straightforward.

**Comment 9:** Line 252: explain here what Schisandrin B (Sch B) is and what it is used for. Not everyone knows.

**Response 9:** Thank you for your valuable feedback. To address your concern, we have made a modification in Line 266-268 to provide a brief explanation of Sch B and its usage. Specifically, we have included the following sentence: "Sch B, an active ingredient from Schisandrae chinensis (Turcz.) Baill. (Schisandraceae) Fructus, has been shown to possess diverse pharmacological effects, such as antitumor, antioxidant, anti-inflammation, and hepatoprotection. " Additionally, we have highlighted this explanation in red to draw your attention to the change.

**Comment 10:** Line 371: "CRS" appears here but it is not explained. At least spell it out.

**Response 10:** Thank you for pointing out the need for clarification regarding the abbreviation "CRS". "CRS" stands for Cytokine Release Syndrome, which is a potentially serious immune reaction that can occur in response to certain therapeutic treatments. As you mentioned, we have already explained this abbreviation in Section 2.5, specifically in Line 203, where we discuss Granzyme-induced Pyroptosis.

**Comment 11:** Line 414: FAD… did you mean FDA?

**Response 11:** Thank you for catching the typo, where "FAD" was incorrectly used instead of "FDA". This was indeed a clerical error that has now been corrected. As mentioned, the mistake has been rectified in Line 432, which is highlighted in red.

Thank you for your valuable feedback on our manuscript. We have undertaken in-depth language polishing, with the revised sections highlighted in yellow, and addressed the typos you mentioned, especially in the conclusion section, to elevate the overall quality. Moreover, we have engaged a professional editing service to conduct a thorough language review, thus ensuring the clarity and accuracy of the manuscript.
